# Chronic TrkB agonist treatment in old age does not mitigate diaphragm neuromuscular dysfunction

**DOI:** 10.14814/phy2.13103

**Published:** 2017-01-13

**Authors:** Sarah M. Greising, Amrit K. Vasdev, Wen‐Zhi Zhan, Gary C. Sieck, Carlos B. Mantilla

**Affiliations:** ^1^ Department of Physiology and Biomedical EngineeringMayo ClinicRochesterMinnesota; ^2^ Anesthesiology and Perioperative MedicineMayo ClinicRochesterMinnesota

**Keywords:** 7‐8‐dihydroxyflavone, Brain‐derived neurotrophic factor, Neuromuscular transmission failure, Tropomyosin‐related kinase

## Abstract

Previously, we found that brain‐derived neurotrophic factor (BDNF) signaling through the high‐affinity tropomyosin‐related kinase receptor subtype B (TrkB) enhances neuromuscular transmission in the diaphragm muscle. However, there is an age‐related loss of this effect of BDNF/TrkB signaling that may contribute to diaphragm muscle sarcopenia (atrophy and force loss). We hypothesized that chronic treatment with 7,8‐dihydroxyflavone (7,8‐DHF), a small molecule BDNF analog and TrkB agonist, will mitigate age‐related diaphragm neuromuscular transmission failure and sarcopenia in old mice. Adult male *Trk* *B*^*F*^ ^*616A*^ mice (*n* = 32) were randomized to the following 6‐month treatment groups: vehicle‐control, 7,8‐DHF, and 7,8‐DHF and 1NMPP1 (an inhibitor of TrkB kinase activity in *Trk* *B*^*F*^ ^*616A*^ mice) cotreatment, beginning at 18 months of age. At 24 months of age, diaphragm neuromuscular transmission failure, muscle‐specific force, and fiber cross‐sectional areas were compared across treatment groups. The results did not support our hypothesis in that chronic 7,8‐DHF treatment did not improve diaphragm neuromuscular transmission or mitigate diaphragm muscle sarcopenia. Taken together, these results do not exclude a role for BDNF/TrkB signaling in aging‐related changes in the diaphragm muscle, but they do not support the use of 7,8‐DHF as a therapeutic agent to mitigate age‐related neuromuscular dysfunction.

## Introduction

Age‐related neuromuscular dysfunction and sarcopenia (i.e., muscle fiber atrophy and loss of force) are evident in the diaphragm muscle (Greising et al. 2013, 2015b, 2015c). Impaired neuromuscular transmission in the diaphragm muscle occurs well before noticeable atrophy or force loss (Greising et al. 2015a). In mice, between young adulthood (6 months of age; 100% survival) and early old age (18 months of age; 90% survival), there is ~30% impairment in diaphragm neuromuscular transmission that persists into old age (24 months of age; 75% survival). A reduction in diaphragm muscle‐specific force (force per cross‐sectional area) is only evident by 24 months of age in mice (Greising et al. 2013, 2015b, 2015c) and rats (Elliott et al. 2016b), and there is atrophy of type IIx and/or IIb fibers in both species (Elliott et al. 2016b). Diaphragm muscle sarcopenia reduces the capacity to generate higher force motor behaviors, including the response to airway occlusion (Mantilla and Sieck 2013; Greising et al. 2015b; Elliott et al. 2016a), consistent with predominant aging effects on the largest and strongest type IIx and/or IIb muscle fibers (Sieck and Fournier 1989; Sieck 1991, 1994; Geiger et al. 2000; Mantilla et al. 2010; Mantilla and Sieck 2011). The mechanisms underlying sarcopenia remain unknown, but age‐related disruption of trophic interactions at the neuromuscular junction are likely to contribute to ongoing denervation, remodeling, and muscle fiber atrophy (Mantilla and Sieck 2009). Previously, we found that denervation induces atrophy that is limited to type IIx and/or IIb muscle fibers in the diaphragm (Miyata et al. 1995b; Zhan et al. 1997; Geiger et al. 2003; Aravamudan et al. 2006).

The signaling of brain‐derived neurotrophic factor (BDNF) through its high‐affinity tropomyosin‐related kinase subtype B (TrkB) receptor plays an important role in maintaining neuromuscular junction structure and neuromuscular transmission (Funakoshi et al. 1995; Mantilla et al. 2004; Mantilla and Ermilov 2012; Greising et al. 2015a). Using a knockin mouse model (*TrkB* ^*F616A*^ mouse), which allows for rapid inhibition of TrkB kinase activity, we showed that inhibition of TrkB kinase activity results in impaired diaphragm muscle neuromuscular transmission (Greising et al. 2015a). Importantly, the effect of TrkB kinase inhibition on diaphragm muscle neuromuscular transmission was lost in older mice. These results suggest that aging may be associated with decreased expression of either BDNF and/or TrkB at the neuromuscular junction. Recently, we showed that the highly selective BDNF analog and TrkB agonist, 7,8‐dihydroxyflavone (7,8‐DHF), acutely improves diaphragm neuromuscular transmission in the diaphragm muscle of young adult mice (Mantilla and Ermilov 2012). In the present study, we hypothesized that chronic 7,8‐DHF treatment would mitigate age‐related diaphragm neuromuscular transmission failure and sarcopenia (atrophy and force loss) in old mice.

## Methods

### Animals

Adult male *TrkB* ^*F616A*^ mice (*n* = 32) at 18 months of age were randomized to vehicle‐control, 7,8‐DHF (highly bioavailable BDNF analog), or cotreatment of 7,8‐DHF and 1NMPP1(inhibiting TrkB kinase activity) and treated until 24 months of age. All mice were genotyped with previously described primers (Mantilla et al. 2014a; Greising et al. 2015a). The *TrkB* ^*F616A*^ mice have a phenylalanine‐to‐alanine mutation in the ATP‐binding domain of the TrkB receptor (Chen et al. 2005), allowing for rapid and selective chemical inhibition of TrkB kinase activity with treatment of 1NMPP1 (Mantilla and Ermilov 2012; Mantilla et al. 2014a, 2014b; Greising et al. 2015a). Specific groups received oral vehicle treatment (0.3% DMSO in drinking water), 7,8‐DHF (5 mg/kg/day; Tocris #3826 (Zhang et al. 2014)), or 7,8‐DHF and 1NMPP1 (25 *μ*mol/L in drinking water; Calbiochem #529581 (Mantilla et al. 2014a, 2014b)). Previously, the inhibition of TrkB kinase activity by oral 1NMPP1 was confirmed in vivo (Mantilla et al. 2014a, 2014b). At the terminal experiment, all mice were anesthetized with an i.p. injection of Fentanyl (0.3 mg/kg), Droperidol (15 mg/kg), and Diazepam (5 mg/kg) and euthanized by exsanguination. All protocols and animal care guidelines were approved by the Institutional Animal Care and Use Committee at the Mayo Clinic, in compliance with National Institute of Health Guidelines.

Mice at 18 and 24 months of age, represent survival rates of 90%, and 75%, respectively, based on data from our colony and published estimates (Turturro et al. 1999; Flurkey 2009; Greising et al. 2013, 2015a). The experimental design accounted for this attrition of animals due to natural aging. Of the 32 mice that were used in this study only 23 completed the 6 month treatment. Of the mice that did not complete the 6 month treatment: one had an abdominal tumor, one had a spleen tumor, two had bowel obstructions/tumors, four died of unknown causes, and one was euthanized per our veterinary staff members due to an ocular infection. Of the 23 mice that completed the 6 month treatment and functional testing at 24 months of age: one had an abdominal tumor, three had bowel obstructions/tumor, and three had liver tumors. Those with liver tumors were excluded from analysis due to anatomical proximity of the liver and diaphragm muscle; this exclusion did not change the results of the study. There was no apparent treatment group effect of the mice presenting with these pathophysiologies, and it is likely that these reflect normal aging effects in the mouse colony. Mice were weighed monthly to monitor any weight loss greater than 10% over the course of the 6 month treatment.

### Diaphragm muscle force and neuromuscular transmission failure

In mice at 24 months of age, diaphragm muscle isometric force and the contribution of neuromuscular transmission failure to diaphragm muscle fatigue were determined as previously described (Kuei et al. 1990; Fournier et al. 1991; Johnson and Sieck 1993; Miyata et al. 1995a; Prakash et al. 1999; Mantilla et al. 2004, 2013, 2014a; Mantilla and Sieck 2009; Ermilov et al. 2010; Mantilla and Ermilov 2012; Sieck et al. 2012; Greising et al. 2013, 2015a, 2015b). Briefly, the diaphragm muscle was dissected with the phrenic nerve intact, and strips (~3 mm width) were cut from the midcostal region of both the left and right diaphragm and attached to a force transducer. All experiments were conducted while the diaphragm muscle was maintained at 26°C and incubated in Reese‐Simpson buffer (pH 7.4) with 95% O_2_ and 5% CO_2_. Muscle length was adjusted until minimal passive force was observed. Isometric force was then evoked by direct muscle stimulation using silver plate electrodes with a 0.5‐ms duration pulse. Stimulus current intensity was increased until maximal twitch force (P_t_) responses were obtained and then set at 125% of this current strength (supramaximal). Muscle length was then adjusted until optimal length for P_t_ was observed (L_o_). Maximum isometric force (P_o_) was evoked at a stimulus rate of 120 Hz in a 1 s duration train. Specific force was calculated by normalizing P_t_ and P_o_ to the cross‐sectional area of the muscle strip determined as diaphragm muscle mass/(optimal length x muscle density). Neuromuscular transmission failure was determined by comparing forces evoked by phrenic nerve stimulation (via a suction electrode) at 40 Hz in 330‐ms duration trains repeated each s for a 2‐min period to force evoked by direct muscle stimulation (via the silver plate electrodes) at 40 Hz in 330‐ms trains that was superimposed every 15 sec. The extent of neuromuscular transmission failure was determined by the difference in force generated by nerve versus direct muscle stimulation as: 100*(NF – MF)/(100 – MF) where NF and MF are the percent decrement in force during nerve and muscle stimulation respectively. For all animals both the right and left diaphragm‐phrenic nerve segments were analyzed due to technical challenges with aging tissue. The results reflect the side that showed the highest levels of neuromuscular transmission. There were no differences in the overall results if right and left segments were averaged across animals.

### Diaphragm muscle fiber type specific atrophy and clustering

Cross‐sectional areas of type‐identified diaphragm muscle fibers were determined as previously described (Sieck et al. 2012; Greising et al. 2013, 2015c). Briefly, strips of diaphragm muscle were cut from the midcostal region adjacent to that used for contractile testing. Length of the diaphragm muscle strip was set to approximate L_o_ (1.5 × resting length), pinned to cork and quickly frozen in melting isopentane. Cross‐sections (10 *μ*m thickness) of the diaphragm muscle were cut using a cryostat, and used for histochemical classification of myosin heavy chain (MyHC) isoforms and fiber cross‐sectional area measurements. Muscle sections were triple labeled with previously validated primary antibodies for MyHC isoforms: anti‐MyHC_Slow_ (Vector Labs VP‐M667) and anti‐MyHC_2A_ (SC‐71 obtained from Developmental Studies Hybridoma Bank [DSHB], Iowa City IA) as well as laminin (Sigma L9393) to label the extracellular matrix surrounding all diaphragm muscle fibers and appropriate fluorescently‐conjugated secondary antibodies (Schiaffino et al. 1989; Zhan et al. 1997; Geiger et al. 1999; Radzyukevich et al. 2013). Each section was imaged by confocal microscopy (Nikon Instruments Inc., Melville, NY) using argon (488 nm) and solid state (405 and 561 nm) lasers for multi‐label fluorescence imaging. Images were saved separately for each fluorescence channel as 16‐bit grayscale‐TIFF files in NIS‐Elements software (Nikon Instruments Inc.) and merged using MetaMorph software (Molecular Devices LLC., Sunnyvale, CA).

Each diaphragm muscle segment was used to determine fiber cross‐sectional area, the average inter‐fiber distance to all fibers, and the average interfiber distance to the 3 and 1 closest fibers using an automated analysis methodology previously described (Greising et al. 2015c). Muscle fibers were classified as type I based on the expression of MyHC_Slow_, as type IIa based on MyHC_2A_ expression, and as type IIx and/or IIb based on the absence of expression of the other two isoforms, permitting all measurements to be performed on a fiber type specific basis. Morphologic analysis was compared to a set of untreated male *TrkB* ^*F616A*^ mice (*n* = 5) that were 24 months of age to confirm the lack of effects due to vehicle‐control treatment.

### Statistical analysis

Data was analyzed using JMP (version 10.0 SAS Institute, Inc., North Carolina). Based on previous studies exploring neuromuscular transmission failure in the diaphragm muscle, an n of 6 per group provides adequate statistical power. Data was analyzed by one‐way ANOVA comparing across treatment group for neuromuscular transmission failure and P_t,_ P_o_, and P_t_/P_o_. Two‐way repeated measures ANOVA was used to compare the extent of neuromuscular transmission failure across age and treatment groups. A mixed linear model, with individual animals as a random effect, was used to analyze fiber type‐specific cross‐sectional areas, normalized distance to the closest fiber and 3‐closest fibers. Tukey‐Kramer's honestly‐significant difference *post hoc* analyses were conducted when appropriate. Data are reported as mean ± SE, unless otherwise specified, significance was accepted at *P* < 0.05.

## Results

### Animal characteristics

At 24 months of age, there were no differences in body mass across vehicle‐control, 7,8‐DHF, or 7,8‐DHF and 1NMPP1 cotreatment groups (Table 1). At the onset of treatment the mean body weight of mice was 41 ± 1 g and at termination 38 ± 1 g. There was no effect of time or treatment group on body mass (*P* = 0.296).

**Table 1:** Group characteristics of all included *TrkB* ^*F616A*^ mice at 24 months of age

	Vehicle‐Control	7,8‐DHF	7,8‐DHF + 1NMPP1	One‐way ANOVA
	(*n* = 8)	(*n* = 6)	(*n* = 9)	*P*‐value
Age (mo)	24.0 ± 0.1	23.9 ± 0.1	23.9 ± 0.1	*0.711*
Body Mass (g)	40.3 ± 3.4	37.7 ± 2.5	35.0 ± 1.5	*0.323*

Data are mean ± SE.

### Diaphragm muscle neuromuscular transmission failure

**Figure 1:**
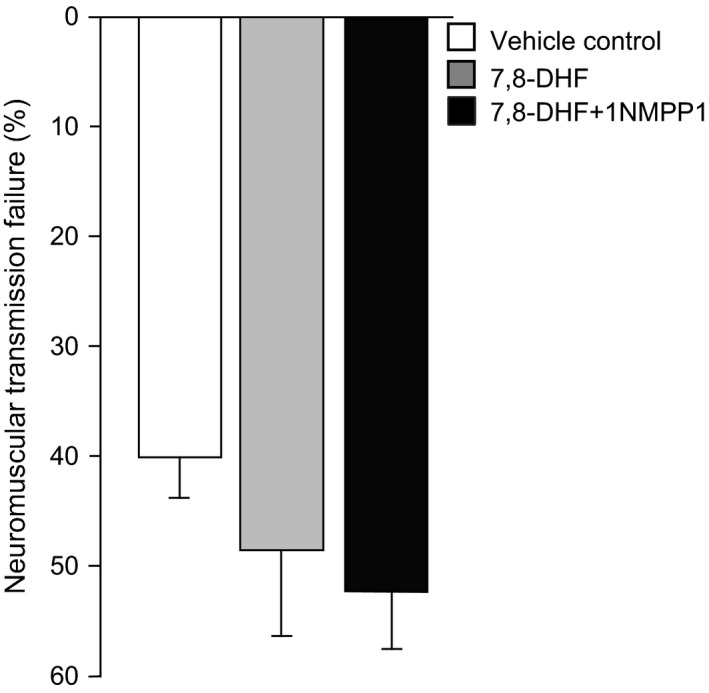
Neuromuscular transmission failure following 2 min of repetitive nerve stimulation and superimposed intermittent muscle stimulation is not affected by a 6 month treatment with vehicle‐control, 7,8‐DHF, or cotreatment with 7,8‐DHF and 1NMPP1 (*P* = 0.278; mean ± SE). Data analyzed by one‐way ANOVA.

The global measure of diaphragm neuromuscular transmission was determined by the analysis of the relative contribution to muscle fatigue over a 2 min period of repetitive nerve (via suction electrode) and intermittent muscle stimulation (via plate electrodes). There was no significant effect of 6 month 7,8‐DHF treatment alone or in combination with 1NMPP1 on diaphragm neuromuscular transmission failure (*P* = 0.278; Fig. 1). Overall across treatment groups, there was a 47.2 ± 5.2% transmission failure after 2‐min of repetitive phrenic nerve stimulation. These results are similar to our previous findings (Greising et al. 2015a), in which diaphragm neuromuscular transmission failure is impaired (~30%) at 24 months compared to 6 months of age. At the onset of repetitive stimulation, there were no differences in the ratio of nerve‐ versus muscle‐evoked forces (Table 2).

**Table 2:** Contractile parameters of the diaphragm muscle following 6‐month treatment

	Vehicle‐Control	7,8‐DHF	7,8‐DHF + 1NMPP1	One‐way ANOVA
	(*n* = 6)	(*n* = 5)	(*n* = 7)	*P*‐value
Pt/Po	0.31 ± 0.03	0.30 ± 0.02	0.29 ± 0.03	*0.876*
Initial Nerve/ Muscle Force (%)	92.8 ± 5.9	79.2 ± 6.4	72.5 ± 5.5	*0.067*

Data are mean ± SE.

### Diaphragm muscle force

**Figure 2:**
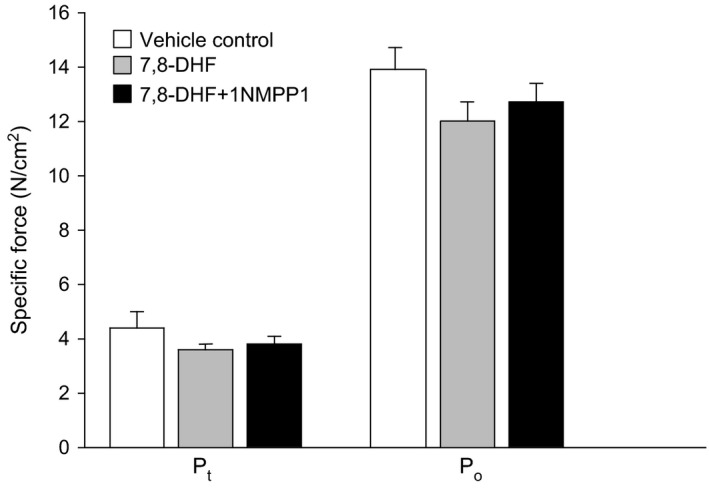
Maximal isometric twitch (P_t_) and tetanic (P_o_) force was normalized to physiological cross‐sectional area of the diaphragm muscle (mean ± SE). There was no effect of treatment group on either contractile parameter for specific P_t_ (*P* = 0.571) or specific P_o_ (*P* = 0.246) following a 6 month treatment of vehicle‐control, 7,8‐DHF, or cotreatment of 7,8‐DHF and 1NMPP1. Data analyzed by one‐way ANOVA.

Consistent with our previous findings (Greising et al. 2013, 2015a, 2015b), diaphragm muscle P_t_ and P_o_ were reduced in mice at 24 months of age (~25% compared to 6 months of age), but there was no effect of either 7,8‐DHF or 7,8‐DHF and 1NMPP1 cotreatment on diaphragm muscle force (*P* ≥ 0.246; Fig. 2). Diaphragm muscle P_t_ was 3.9 ± 0.5 N/cm^2^ and P_o_ was 12.6 ± 0.7 N/cm^2^ averaged across all treatment groups (Fig. 2). There was no difference in P_t_/P_o_ across treatment groups (Table 2).

### Diaphragm muscle morphology

Since, neither 7,8‐DHF alone or 7,8‐DHF and 1NMPP1 cotreatment impacted diaphragm muscle force, we compared muscle morphology only between vehicle‐control and 7,8‐DHF groups. Additionally, to account for any differences in chronic DMSO administration, we compared muscle morphology results from vehicle‐control and 7,8‐DHF groups to untreated 24 month *TrkB* ^*F616A*^ mice.

Consistent with previous reports (Greising et al. 2013, 2015c), there were differences in cross‐sectional area across muscle fiber types (*P* < 0.001; Table 3), specifically there was an expected age‐related atrophy of the IIx and/or IIb diaphragm muscle fibers (~25% compared to 6 months of age). There was no effect of treatment on the cross‐sectional area of diaphragm muscle fibers (*P* = 0.180). Although there was a significant interaction between fiber type and treatment group (*P* < 0.001), there was no difference in the cross‐sectional areas of type I, type IIa, or type IIx and/or IIb diaphragm muscle fibers across treatment groups (Table 3). Cross‐sectional areas of type IIx and/or IIb fibers were significantly larger than type I and type IIa diaphragm muscle fibers across treatment groups. In the 7,8‐DHF treatment group, type I fibers were also significantly smaller than type IIa fibers. There was no difference in fiber type proportions in the diaphragm muscle across treatment groups (*P* = 0.266).

**Table 3:** Diaphragm muscle fiber cross‐sectional areas in old mice (*μ*m^b^)

Animal (n)	Untreated	Vehicle‐Control	7,8‐DHF	Mixed Linear Model *P‐*value		
	(*n* = 5)	(*n* = 6)	(*n* = 6)	Fiber Type effect	Treatment effect	Interaction
Type I^a^	542.2 ± 61.6 (218)	669.7 ± 62.4 (182)	496.1 ± 62.6 (175)	*<0.001*	*0.180*	*<0.001*
Type IIa^b^	579.6 ± 57.2 (1235)	857.7 ± 57.3 (984)	711.1 ± 57.3 (958)			
Type IIx and/or IIb	793.7 ± 60.1 (321)	859.2 ± 57.7 (737)	888.8 ± 58.5 (482)			

Data are least squares mean ± SE in *μ*m^2^. The number of muscle fibers of each type are in parenthesis.

a Significant main effect of fiber type; different than type IIa and type IIx and/or IIb fibers.

b Significant main effect of fiber type; different than type IIx and/or IIb fibers.

Diaphragm muscle fibers are arranged in a hexagonal array with each fiber surrounded by six fibers. To determine if 7,8‐DHF treatment had an effect on spatial patterns or clustering of MyHC fibers (indicating neuromuscular junction denervation) various parameters were analyzed, including the average interfiber distance and the distance to the 1‐ and 3‐closest fibers both for all fibers and in a fiber type‐specific analysis. Interfiber distances were also normalized by the average interfiber distance for all fibers in order to account for potential differences across animals. There was no effect of treatment group on the normalized type‐specific interfiber distance to the 1‐ and 3‐closest fibers (*P* > 0.544; Table 4). However, as expected, there was a main effect of fiber type (*P* < 0.001). The normalized interfiber distance to both the 1‐ and 3‐clostest fibers was greatest in all type I fibers, regardless of treatment group (*P* < 0.001; Table 4), consistent with previous results (Greising et al. 2015c).

**Table 4:** Fiber type dependent average interfiber distances (mm)

	Untreated	Vehicle‐Control	7,8‐DHF	Mixed Linear Model *P‐*value		
	(*n* = 5)	(*n* = 6)	(*n* = 6)	Fiber Type effect	Treatment effect	Interaction
Normalized Distance to the Closest Fiber						
Type I^a^	0.25 ± 0.13	0.34 ± 0.12	0.39 ± 0.12	*<0.001*	*0.467*	*0.295*
Type IIa	0.06 ± 0.12	0.19 ± 0.12	0.31 ± 0.12			
Type IIx and/or IIb	0.09 ± 0.13	0.21 ± 0.12	0.37 ± 0.12			
Normalized Distance to 3‐Closest Fibers						
Type I^a^	0.19 ± 0.02	0.25 ± 0.02	0.25 ± 0.02	*<0.001*	*0.664*	*<0.001*
Type IIa	0.09 ± 0.02	0.11 ± 0.02	0.10 ± 0.02			
Type IIx and/or IIb	0.13 ± 0.02	0.11 ± 0.02	0.13 ± 0.02			

Data are least squares mean ± SE. Distances represent average interfiber distance for fibers of the same type.

a Significant main effect of fiber type; different than type IIa and type IIx and/or IIb fibers.

## Discussion

Treatment with the small molecule 7,8‐DHF was investigated as a possible therapeutic option to counter age‐related neuromuscular dysfunction through systemic targeting of BDNF/TrkB signaling. Contrary to our hypothesis that enhancing BDNF/TrkB signaling with 7,8‐DHF would mitigate age‐related neuromuscular dysfunction, we found no effect of 7,8‐DHF treatment on diaphragm neuromuscular transmission failure, muscle specific force, fiber cross‐sectional areas or fiber type clustering, a surrogate marker for denervation. Collectively, these results indicate that enhancing BDNF/TrkB signaling with 7,8‐DHF delivered chronically starting at an early old age (18 months of age) is not effective to mitigate sarcopenia in the diaphragm muscle.

### 7,8‐dihydroxyflavone is a small molecule TrkB agonist

The TrkB agonist 7,8‐DHF has recently gained attention as being advantageous in a variety of age‐related conditions including stroke, depression, memory and learning, traumatic brain injury, Rett syndrome, Amyotrophic Lateral Sclerosis, and Parkinson's Disease (Obianyo and Ye 2013; Jang et al. 2010; Andero et al. 2011). In particular, 7,8‐DHF shows high bioavailability with systemic delivery and high affinity for the TrkB receptor, mimicking many positive BDNF effects. The pharmacokinetic profile of 7,8‐DHF is now well characterized, with bioavailability of 5% after oral administration and a half‐life of 134 min in the plasma (Liu et al. 2013; Zhang et al. 2014). Indeed, 7,8‐DHF shows therapeutic efficacy when delivered chronically in animal models of depression (Liu et al. 2013), Alzheimer's disease (Zhang et al. 2014) and other neurodegenerative diseases (Jang et al. 2010). We previously showed that acute 7,8‐DHF treatment improved diaphragm neuromuscular transmission failure induced by repetitive phrenic nerve stimulation in adult mice (Mantilla and Ermilov 2012), with effects similar to those previously shown for BDNF in adult rats (Mantilla et al. 2004) and mice (Greising et al. 2015a). For both 7,8‐DHF and BDNF treatment, effects were shown to be mediated by TrkB activation since inhibition of TrkB kinase activity with 1NMPP1 in *TrkB* ^*F616A*^ mice and by the kinase inhibitor K252a in rats blunted these positive effects. Impairments in neuromuscular transmission are an important, early feature of diaphragm motor dysfunction in old age (Greising et al. 2015a). By mimicking the neurotrophic activity of BDNF on neuromuscular transmission, we hypothesized that 7,8‐DHF should be effective in mitigating age‐related changes at the diaphragm muscle.

### BDNF/TrkB signaling is disrupted in old age

There is converging evidence that loss of neurotrophic activity in old age may contribute to neuromuscular dysfunction, which could contribute to other aging effects on the neuromuscular system including sarcopenia. In a previous study (Greising et al. 2015a), we reported that age determines the effects of BDNF on neuromuscular transmission. While BDNF mitigates diaphragm neuromuscular transmission failure with repetitive activation in young adult mice (6 months of age) and in early old age (18 months of age), BDNF is no longer effective in older animals (24 months of age). Importantly, the age‐related impairment in neuromuscular transmission (~30% between 6 and 24 months of age) is similar to the effect of acute inhibition of TrkB kinase activity in young adult mice. Furthermore, inhibiting TrkB kinase activity for 7 days (with 1NMPP1 treatment in *TrkB* ^*F616A*^ mice) resulted in significant impairment of diaphragm neuromuscular transmission in 6 and 18 month old mice (Mantilla et al. 2014b; Greising et al. 2015d). It is worth noting that the effects of BDNF/TrkB signaling at the neuromuscular junction are primarily presynaptic and involve modulation of synaptic vesicle release (Lohof et al. 1993; Lu 2004; Mantilla et al. 2004, 2014b; Garcia et al. 2010; Greising et al. 2015a). In young adult mice, 7‐day inhibition of TrkB kinase activity resulted in more compact, less fragmented motor end‐plates, and small differences in presynaptic terminal volume (~20%) and motor end plate area (~10%). These changes recapitulate several features evident at diaphragm neuromuscular junctions in older animals, including an age‐related reduction in the proportion of large neuromuscular junctions. Changes in neuromuscular junction structure and function are similar to those in old age when expression of the TrkB receptor is genetically reduced (*TrkB* ^*+/−*^ mouse) (Kulakowski et al. 2011). Importantly, following 7‐day inhibition of TrkB kinase activity in early old age, there was evidence of reduced overlap of pre‐ and postsynaptic structures at diaphragm neuromuscular junctions and an increased proportion of denervated muscle fibers (~20% compared to young adult mice). In very old mice and rats, fragmentation and denervation of motor end‐plates in the diaphragm muscle is significant (Prakash and Sieck 1998; Valdez et al. 2012) and these changes are consistent with findings in other muscles including rodent hindlimbs (Cardasis and LaFontaine 1987; Deschenes et al. 2010).

### BDNF/TrkB signaling maintains structural and functional integrity at the neuromuscular junction

The importance of BDNF/TrkB signaling in maintaining structural and functional properties of neuromuscular junctions is also revealed by the reversibility of effects. Indeed, inhibition of TrkB kinase activity exerts largely reversible effects on neuromuscular dysfunction in adult mice (Mantilla et al. 2014b). Following withdrawal of the TrkB kinase inhibitor in 1NMPP1‐treated *TrkB* ^*F616A*^ mice, neuromuscular transmission was restored. Furthermore, when 1NMPP1 treatment was withdrawn for 7 days, recovery of TrkB kinase activity is sufficient to restore presynaptic terminal and motor end‐plate morphology. Collectively, available evidence suggests that aging is associated with reduced BDNF/TrkB signaling at neuromuscular junctions and that this aging effect may be reversible by exogenous treatment with TrkB agonists and restored TrkB kinase activity. The lack of effects on neuromuscular dysfunction by the TrkB agonist 7,8‐DHF in the present study may reflect reduced TrkB receptor availability into old age that is not mitigated by enhancing agonist availability. It is tempting to speculate that increasing TrkB receptor presynaptically may be adequate to improve BDNF/TrkB signaling and diaphragm neuromuscular dysfunction. Indeed, we recently increased TrkB receptor expression in phrenic motor neurons using a gene therapy approach based on viral transduction (Gransee et al. 2012; Martinez‐Galvez et al. 2016). However, this approach resulted in increased TrkB expression in a relatively small number of motor neurons (~15%). Novel developments in viral technology would be needed to improve effectiveness and transduction to motor neurons in order to envision robust mitigation of aging effects with such therapy. It is clear that TrkB agonist administration does not prevent the long‐term, age‐related effects at the neuromuscular junction even though acute effects are present. In addition, this time course of aging effects whereby reduced BDNF availability precedes loss of TrkB receptor availability and the appearance of sarcopenia suggests a window of susceptibility to aging effects on the neuromuscular system and is of specific interest, warranting future investigation.

### Diaphragm muscle sarcopenia

Age‐related disruption at the neuromuscular junction and impaired neuromuscular transmission may result in muscle disuse and decreased trophic influences on the muscle, impairing trophic interactions between motor neurons and muscle fibers (Greising et al. 2015a, 2015c). Collectively, these factors may contribute to sarcopenia, which is associated with selective type IIx and/or IIb fiber atrophy. In previous studies, unilateral denervation of the diaphragm muscle induced muscle fiber atrophy also limited to type IIx and/or IIb fibers (Miyata et al. 1995b; Zhan et al. 1997; Geiger et al. 2003; Aravamudan et al. 2006). These fiber types comprise the most fatigable, yet strongest motor units and the loss of their contribution would have a greater overall impact on force loss (Mantilla and Sieck 2013). Specifically, sarcopenia in the diaphragm muscle of mice is characterized by type IIx and/or IIb muscle fiber atrophy and an ~25% reduction in specific force (Greising et al. 2013, 2015a), with an associated reduction in the capacity to generate higher‐force motor behaviors such as during tracheal occlusion (Greising et al. 2015b). These sarcopenia effects are not evident in early old age (18 months of age) and become evident only in old age (24 months of age), which justified starting 7,8‐DHF treatment at 18 months in the present study. The results of the present study confirmed the reduction in type IIx and/or IIb diaphragm muscle fiber cross‐sectional areas and specific force in mice at ~24 months of age (Greising et al. 2013, 2015c). The cross‐sectional areas of type I and IIa fibers did not change with age. Consistent with the lack of an effect of chronic treatment with 7,8‐DHF on neuromuscular transmission, there was no effect on diaphragm muscle force or fiber type cross‐sectional areas at 24 months of age. There was also no evidence of a change in the neuromuscular junction denervation (using the surrogate of muscle fiber type clustering) in the current study.

## Conclusions

The interaction between aging effects and disruption in BDNF/TrkB signaling at the neuromuscular junction is clearly complex. Although there is physiological evidence of reduced BDNF availability in early old age (Greising et al. 2015a), measurements of BDNF release at individual neuromuscular junctions have not been conducted. A previous report in the rat hippocampus and prefrontal cortex documented age‐related reductions in BDNF, albeit with somewhat variable transcriptional and translational impairments (Calabrese et al. 2013). The current study did not investigate BDNF availability or TrkB receptor expression or signaling within the neuromuscular system. Understanding changes in the expression and downstream signaling of the TrkB receptor expression with advanced age is also important and should be further investigated. Available evidence supports a role for neurotrophins in regulating their own expression as well as expression of their receptors in a positive‐feedback fashion. For example, studies using PC12 and cultured neocortical cells show that neurotrophins can increase expression of other neurotrophins (Canossa et al. 1997; Kruttgen et al. 1998; Xiong et al. 2002), as well as upregulate their receptors (Rankin et al. 2005, 2008). Based on the results of the present study, this positive‐feedback effect does not seem to be reproduced in vivo, at least with chronic treatment starting at 18 months of age in mice. In this sense, it is worth recognizing that future research should focus on the quality and expression of the TrkB receptor in old age. Additional therapeutic options to enhance and/or maintain TrkB receptor expression (e.g., using gene therapy approaches) at the neuromuscular junction could be explored in order to provide not only increased TrkB agonist availability but also increased TrkB receptor expression and possibly mitigate age‐related neuromuscular dysfunction. Lastly, chronic treatment with the TrkB agonist 7,8‐DHF was started at an age in which neuromuscular impairments are already evident but when force loss is not yet evident, and it is possible that TrkB agonist treatment that began at an even earlier age is necessary.

## Conflict of Interest

The authors declare no conflict of interest.
